# Characterization of Aptamer-Protein Complexes by X-ray Crystallography and Alternative Approaches

**DOI:** 10.3390/ijms130810537

**Published:** 2012-08-22

**Authors:** Vincent J. B. Ruigrok, Mark Levisson, Johan Hekelaar, Hauke Smidt, Bauke W. Dijkstra, John van der Oost

**Affiliations:** 1 Laboratory of Microbiology, Wageningen University, Dreijenplein 10, Wageningen 6703 HB, The Netherlands; E-Mails: mark.levisson@wur.nl (M.L.); hauke.smidt@wur.nl (H.S.); 2 Laboratory of Biophysical Chemistry, University of Groningen, Nijenborgh 7, Groningen 9747 AG, The Netherlands; E-Mails: j.hekelaar@rug.nl (J.H.); b.w.dijkstra@rug.nl (B.W.D.)

**Keywords:** X-ray crystallography, aptamer, interaction, RNA/DNA-protein complex

## Abstract

Aptamers are oligonucleotide ligands, either RNA or ssDNA, selected for high-affinity binding to molecular targets, such as small organic molecules, proteins or whole microorganisms. While reports of new aptamers are numerous, characterization of their specific interaction is often restricted to the affinity of binding (*K*_D_). Over the years, crystal structures of aptamer-protein complexes have only scarcely become available. Here we describe some relevant technical issues about the process of crystallizing aptamer-protein complexes and highlight some biochemical details on the molecular basis of selected aptamer-protein interactions. In addition, alternative experimental and computational approaches are discussed to study aptamer-protein interactions.

## 1. Introduction

Aptamers are single-stranded DNA (ssDNA) or RNA oligonucleotides selected to bind specifically to a predefined target. Since their initial development two decades ago [1,2] the field of aptamer research has matured. During these years, numerous aptamers, recognizing a wide range of targets, have become available, such as, for example, those binding small organic molecules [3–5], peptides and proteins [6,7], or even whole microorganisms [8,9].

The systematic evolution of ligands by exponential enrichment (SELEX) is the most common method by which aptamers are enriched from large pools of randomized DNA or RNA (10^14^–10^15^ variants), in an iterative process by applying several subsequent selection rounds. Selection is initiated by exposing the oligonucleotides to a target that is either coupled to a matrix (e.g., magnetic beads or column material) or already present on a surface (e.g., cell surface). Subsequently, non-binding oligonucleotide molecules are washed away, and the bound molecules are recovered, amplified by PCR or RT-PCR, and made single-stranded again. Several variations of the SELEX procedure have been successfully applied over the years [10].

In many cases, the minimal sequence required for efficient target binding is smaller than the length of the oligonucleotides that comprise the pool. In order to increase specificity and to reduce synthesis costs of the selected aptamers, the minimal binding sequence should be identified. Approaches to identify the minimal binding sequence can be straightforward when a conserved nucleotide motif or secondary structure is enriched [11,12]; otherwise, identifying the minimal binding sequence can be more laborious [13–15].

Aptamer-target interactions depend on the nature of the target and on the nucleotide sequence and 3D structure of the aptamer. Aptamers occur in a wide variety of structural shapes, such as hairpins, bulges, pseudoknots and G-quadruplexes [10]. Due to these various structural shapes, aptamers can bind their targets by hydrogen bonds, hydrophobic interactions, van der Waals interactions, aromatic stacking or, in most cases, a combination thereof [16].

Crystal structures of aptamer-target complexes provide very detailed information on the interactions; they are therefore crucial for a thorough understanding of the aptamer-target binding mode. However, obtaining crystal structures of aptamer-target complexes has proven difficult, and only a few co-crystal structures have become available over the years (Table 1). Besides X-ray crystallography, also other techniques, such as nuclear magnetic resonance (NMR), surface plasmon resonance (SPR), analysis using a quartz crystal microbalance (QCM) [17], isothermal titration calorimetry (ITC), Dynamic light scattering (DLS), circular dichroism (CD) [18] and small-angle X-ray scattering (SAXS) [12,19], have been used to study aptamer-target binding. Knowledge on the target-binding mode of newly described aptamers, however, is generally restricted to the affinity of binding.

In this review, relevant technical issues and difficulties in the process of crystallizing aptamer-protein complexes are described. In addition, we will highlight some successful examples. Besides X-ray crystallography, several alternative approaches to investigate the molecular basis of aptamer-target interactions will be briefly discussed as well.

## 2. Structure Determination

Currently, the three-dimensional structures of many proteins, nucleic acids (e.g., riboswitches) and other biological molecules have been determined. The only methods that can provide atomic resolution structures are NMR spectroscopy, electron microscopy (electron crystallography) of two-dimensional (2D) crystals, and X-ray crystallography. Each of these methods has its advantages and limitations. In particular, NMR methods for determining high-resolution structures are largely limited to relatively small molecules (<30–40 kDa), due to complexity of the data [36,37]. By comparison, X-ray crystallography can solve structures of arbitrarily large molecules, but it requires crystals that provide suitable quality diffraction data. Screening for conditions that yield well diffracting crystals is still a trial and error method and often requires a significant amount of effort [38]. The chance of obtaining well-ordered 2D crystals for electron crystallography is higher, when compared to obtaining 3D crystals required for X-ray crystallography; however, structure determination and data processing from 2D crystals is still labor intensive and time consuming [39].

While providing very detailed information, crystal structures of aptamer-protein complexes have only scarcely become available. Here, we would like to describe some relevant technical issues in the process of crystallizing aptamer-protein complexes and highlight those parameters that make crystallization especially challenging.

### 2.1. Nucleic Acid Parameters

Several features of DNA or RNA play important roles in the process of co-crystallization. Apart from purity, the length and the ends (blunt or sticky) are also factors that must be explored when crystallizing nucleic acid-protein complexes.

Perhaps one of the most significant variables is the length of the nucleic acid. A general rule in protein-nucleic acid crystallization is to identify a sequence of minimal length that binds tightly to the protein [40,41]. On the one hand, oligonucleotides that are too short will destabilize the complex, as it might limit the number of potential interactions between nucleic acid and protein, but on the other hand it may improve crystal quality, as it will reduce flanking regions that could disturb crystal contacts. Consequently, determining the minimal binding sequence of an aptamer should be considered essential. Although this is not always straightforward and can be time consuming, removing nucleotides that are unnecessary for target binding will prevent them from potentially disturbing crystal contacts.

**Figure 1:**
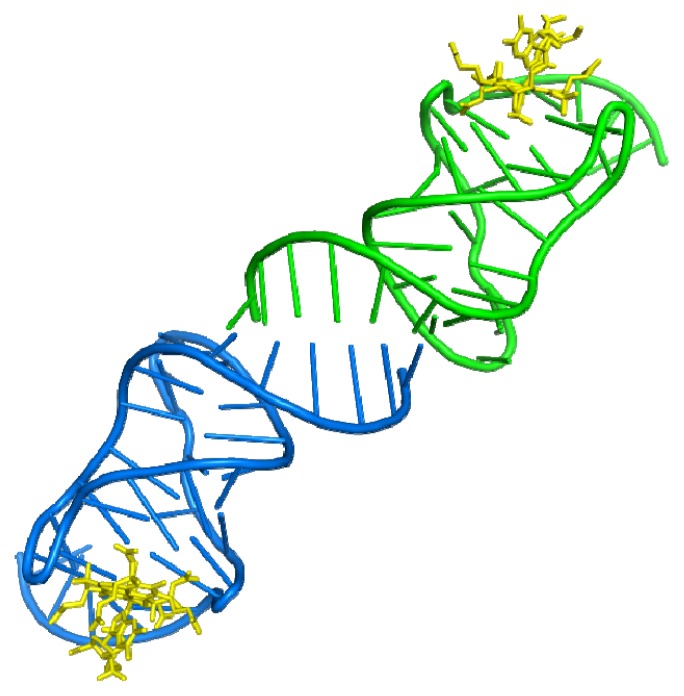
Crystal contacts between two vitamin B_12_ aptamers. The six-base-pair duplex between two vitamin B12 aptamers consists of two stacked adenosine–adenosine pairs (1ET4) [25]. Aptamers shown in blue and green, vitamin B_12_ in yellow.

In addition to blunt-ended nucleic acids, single- or double-base overhangs (sticky ends) are commonly explored when crystallizing complexes containing DNA or RNA [40]. It is often observed in crystals that sticky ends form crystal contacts by base-paring with complementary sticky ends, forming a pseudo-continuous double helix. In order to allow the best end-to-end packing, the overhanging bases of one strand should be complementary to the overhanging bases of the opposite strand [41]. Nevertheless, this may not be easily applicable to aptamers, because sticky ends could disrupt the tertiary structure, and hence abolish binding, of the aptamer. It has been observed, however, that the 5′ extensions from two molecules of the vitamin B_12_ RNA aptamer form a crystal contact by creating a six-base-pair duplex with two stacked adenosine–adenosine pairs [33] (Figure 1).

It may also be critical to have highly purified DNA or RNA to obtain well-ordered crystals of a protein-nucleic acid complex [41]. The most common methods for purifying synthesized oligonucleotides for use in crystallization experiments are anion exchange chromatography and purification from polyacrylamide gels (PAGE). Because results of purification can vary slightly, it is sometimes desirable to further treat the purified oligonucleotide, for instance by dialyzing against an appropriate buffer.

RNA synthesis is relatively expensive; if no modified nucleotides are required, it could be cheaper and perhaps more convenient to transcribe the RNA from a DNA template. When more stable RNAs are required, one could order chemically synthesized RNA aptamers, in which all or specific uridines and cytidines are 2′-fluoro-modified. In some cases, these modified nucleotides have been used during both aptamer selection and crystallization; e.g., [23,42].

### 2.2. Protein Parameters

A general requirement for crystallization experiments is that the protein needs to be homogeneous, *i.e.*, highly pure (>98%) and in a low polydispersity state [43]. DLS is a commonly used technique to measure the polydispersity of a protein sample. Other parameters that should be established before setting up crystallization screens are the stability of the protein in different buffers, and whether the protein is correctly folded (CD spectroscopy) and active (activity measurements).

### 2.3. Protein: Aptamer Ratio

It is good practice to try several protein:aptamer molar ratios. In crystallization experiments, protein and DNA are usually mixed at 1:1.2 to 1:1.5 molar ratios with DNA in excess [41,43]. The principle behind this ratio is that the concentrations of protein and DNA are frequently rough estimations and DNA could possibly not saturate all binding sites on the protein. Our advice is, therefore, to use similar ratios when setting up crystallization experiments of protein-aptamer complexes.

### 2.4. Crystallization Screens

Screens for obtaining crystals should include a wide variety of crystallization conditions. Nowadays, many commercial screens are available from companies such as Hampton Research, Emerald BioSystems, Molecular Dimensions, Jena Bioscience and Qiagen; these also include screens specific for crystallizing nucleic acids and nucleic acid complexes. The commercial screens are undoubtedly the easiest way to initiate the first crystallization trials. For more elaborate information on protein crystallization, see [38,43,44]. For more elaborate information on RNA and sample preparation for crystallization of RNA and RNA-protein complexes in particular, see [45–47].

In general, for nucleic acid crystallization, it is favorable to use polyethylene glycol (PEG) or 2-methyl-2,4-pentanediol (MPD) as precipitants, rather than high salt, because high salt may disrupt charged interactions between protein and nucleic acid. It is not possible to predict which screen will result in crystal formation, because many factors influence crystallization; variables include pH, ionic strength, temperature, protein concentration, the presence of various salts, ligands or additives, the type of precipitant and the crystallization method (hanging drop, sitting drop, dialysis, *etc*.) [38,44].

## 3. Examples from Literature

Over the years, only a limited number of crystal structures of aptamer-protein complexes have become available (Table 1), but they have provided a wealth of information. Here two aptamer-protein complexes are described that have been successfully crystallized.

### 3.1. Aptamer-Thrombin Complex

**Figure 2:**
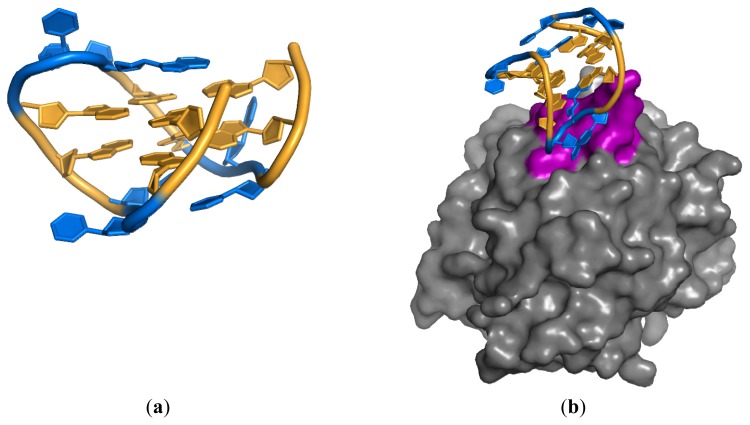
(**a**) Double G-quadruplex shown in gold, loops in blue; (**b**) Thrombin aptamer bound to exosite 1 (magenta) of thrombin (1HUT) [32].

Thrombin is a trypsin-like serine protease with an important role in hemostasis, by converting soluble fibrinogen into insoluble fibrin strands. Shortly after the initial development of SELEX, thrombin-binding DNA aptamers were described [48], providing the first example of DNA binding to a protein normally not involved in DNA binding. The year after thrombin binding aptamers had first been described, a crystal structure of the thrombin-aptamer complex became available. It was obtained using a reservoir solution of 25%–30% (*v*/*v*) PEG 8000, 375 mM NaCl, 0.5 mM NaN_3_ and 50 mM sodium phosphate at pH 7.3, which was also used for the crystallization of a thrombin-hirugen complex [21,49]. The crystal used for data collection grew in 2 months and diffracted to about 2.9 Å, it belongs to orthorhombic space group *P*2_1_2_1_2_1._ In the same year, an NMR solution structure [50,51] became available for the thrombin-aptamer complex as well. The crystal structure and NMR solution structure revealed that the core of the thrombin-binding aptamers is formed by two stacked G-quadruplexes (Figure 2a), although the crystal and NMR solution structure show different topologies of the G-quadruplex [52]. It was also shown that the aptamer binds to exosite I of thrombin (Figure 2b). Recently, another crystal structure of a thrombin-aptamer complex has been published (crystallized at 20 °C using 20% (w/v) PEG 20,000, 200 mM ammonium sulfate, 3% (*v*/*v*) *n*-propanol, 100 M sodium acetate at pH 5.8). It contains a modified thrombin binding aptamer that binds thrombin with higher affinity [24].

### 3.2. Anti-IgG Fc RNA Aptamer

**Figure 3:**
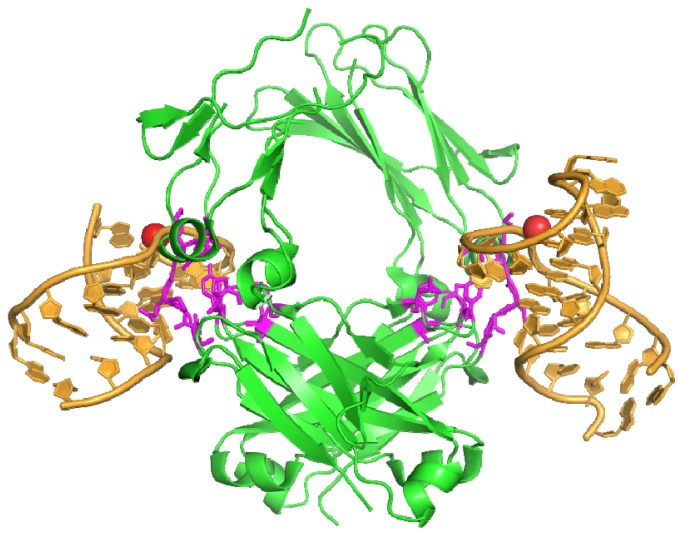
Two RNA aptamers (gold) bound to the Fc domain of human IgG. Residues binding the aptamer are shown in magenta, calcium ions are shown as red spheres (3AGV) [37].

Recently, the 2.15 Å resolution crystal structure of the anti-Fc RNA aptamer in complex with the Fc fragment of a human IgG1 antibody (hFc1) was elucidated [28]. In most cases, RNA aptamers bind to their target proteins predominantly via electrostatic interactions between the negatively-charged phosphate backbones and positively-charged surface residues of proteins. To this end, the interaction between this aptamer and hFc1 is an exception, as it mainly consists of van der Waals contacts and hydrogen bonds, rather than electrostatic forces. The structure also revealed other interesting features. The RNA structure in the complex diverges greatly from its predicted secondary structure; it changes its conformation to one that structurally fits to hFc1 (Figure 3). The aptamer–hFc1 interaction is stabilized by a calcium ion and, therefore, binding and release of the aptamer can be controlled by either chelation or addition of calcium.

Crystals of the RNA aptamer in complex with hFc1 were grown by sitting-drop vapor diffusion using a reservoir solution containing 0.1 M Tris–HCl buffer (pH 8.0), 20% (*w*/*v*) PEG 1000 and 0.2 M calcium acetate. [42]. The RNA aptamer was chemically synthesized containing 2′-fluoropyrimidines and purified by PAGE. NMR analysis showed that the interaction between the Fc fragment and the aptamer has a 1:2 (Fc fragment:aptamer) stoichiometry [53]. Consequently, the aptamer was mixed with the Fc fragment in a molar ratio of 1:2.2 (Fc fragment:aptamer) for crystallization. The crystals belong to the space group *P*2_1_2_1_2 and diffracted to 2.15 Å. Interestingly, crystal quality was improved by applying a solution stirring technique [54].

## 4. Crystallization of a Streptavidin-Aptamer Complex

In previous work, we described the kinetic and stoichiometric characterization of streptavidin-binding aptamers [12]. In order to gain more insight into the molecular basis of the streptavidin-aptamer interaction, crystallization screens of the complex were conducted. Here, as an example, we will briefly describe our crystallization trials and the results obtained.

**Figure 4:**
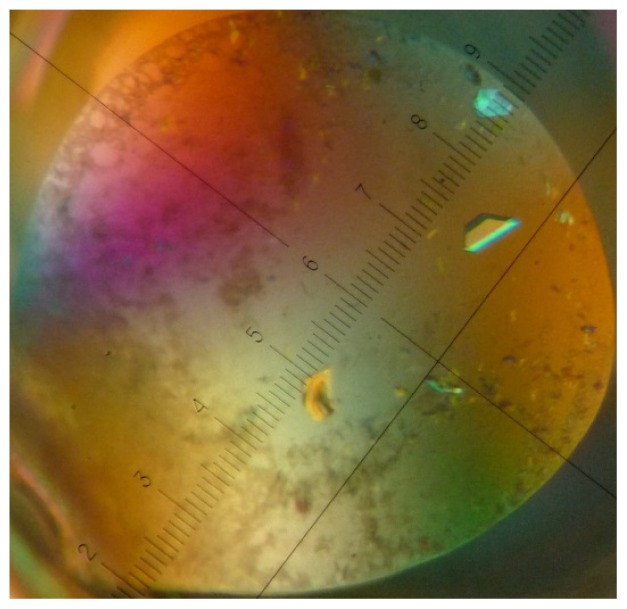
Crystal used for data collection. The crystal was obtained by vapor diffusion against a reservoir solution consisting of 2.0 M ammonium sulfate and 5% (v/v) 2-propanol (Crystal screen 2 condition 5).

For our initial crystallization experiments, we used the commercially available crystallization screens JCSG+ Suite (Qiagen) and Crystal Screen & Crystal Screen 2 (Hampton Research, CA, USA). Streptavidin and aptamer (Figure S1) were mixed in a ratio of 1 streptavidin tetramer to 5 aptamers. For further experimental details, see the Supplementary text. Crystals of sufficient quality for data collection (Figure 4) were obtained from a high salt condition (2.0 M ammonium sulfate and 5% (*v*/*v*) 2-propanol), not containing PEG or MPD. Analysis of the 1.9 Å resolution structure showed the presence of extra electron density in the biotin-binding pocket, but this density was too small to have originated from an aptamer (Figures S2 and S3, for refinement statistics see Table S1).

In a second series of crystallization experiments, we used the Natrix & Natrix 2 screens (Hampton Research, CA, USA), which contain conditions more dedicated towards crystallization of nucleic acids and protein-nucleic acid complexes. Streptavidin and aptamer were mixed in a ratio of 1 tetramer to 2.2 aptamers on the basis of mass-spectrometry [12]. Although recently the use of chromophoric ligands was proposed to visually screen co-crystals of putative protein-nucleic acid complexes [55], we choose to conduct the crystallization screens in triplicate, using (i) streptavidin-aptamer complex, (ii) only streptavidin and (iii) only aptamer. In four conditions, crystals were obtained for both the streptavidin-aptamer complex and streptavidin alone. Crystals of the complex differed largely in size and shape from those of streptavidin only (Table S2), whereas no crystals grew in the same conditions containing only aptamer (pictures not shown). This indicates that the aptamer has a major effect on the crystallization process. Unfortunately, none of the crystals from the streptavidin-aptamer mix were suitable for data collection; they were either microcrystalline needles (1D) or plates (2D), and therefore it remains to be established whether they contain aptamer or not.

## 5. Alternative Methods of Investigating Aptamer Target Interactions

Besides X-ray crystallography, alternative techniques exist to investigate the molecular basis of aptamer-target interactions. When used individually, these methods do perhaps not provide as much detailed information as a crystal structure, and they are probably not as visually appealing. However, using a combination of complementary techniques may still provide very detailed information on the interaction. Together, these techniques could be considered an alternative to X-ray crystallography, for instance, when crystallization trials fail to produce well diffracting crystals, or when no adequate equipment or expertise is available to conduct crystallization trials. Moreover, some of these techniques may provide information on the dynamics of complex formation, whereas crystallography gives a time- and position-averaged image. Since bioinformatics and experimental approaches are generally complementary, both will be discussed.

### 5.1. Computational Approaches

The sequence of an oligonucleotide, and the intramolecular base-pairing that this sequence enables, largely defines the 3D-structure of the oligonucleotide. Base-paired regions in the 3D-structure of the oligonucleotide are thought to act as stabilizers, allowing loops and bulges to position themselves in ways suitable for target interaction [56]. The nucleotide composition of distinct aptamers that bind the same target can vary strongly, particularly in the base-paired regions; however, the overall structure could remain largely similar [11,12,57]. In other words, structural elements shared between oligonucleotides might therefore not be easily visible from primary sequence alignments. In addition, defining the boundaries of the minimal sequence required for target binding is not straightforward. For these reasons, identifying a recurring structure, within a number of sequenced oligonucleotides of an enriched pool, can be challenging if only sequence alignments are used.

Secondary structure predictions of clones in enriched pools may reveal recurring structural features, at any position and with deviating primary structures of oligonucleotides. These predictions could also be useful tools for identifying the minimal binding sequence. Most popular secondary structure prediction programs, including mfold [58], may be of limited value as they can only predict relatively simple hairpin structures. For the analysis of more complex aptamer structures, dedicated programs should be used, e.g., for the prediction of pseudoknots [59] and G-quadruplexes [60].

Besides secondary structure prediction, software has been developed to predict tertiary structures, varying from *ab initio* modeling to approaches requiring detailed information on base pairing and other interactions [61]. Such software has not frequently been used in aptamer research, partly because these programs often do not provide a single prediction, but rather give numerous possibilities that each have to be scored for their relevance. For example, the MC-Fold and MC-Sym pipeline [62] that we have previously used for the generation of a 3D-model of a streptavidin binding aptamer, provided 57 widely different structural models [12]. Eventually a prediction was selected by comparing theoretical scatter of the predicted models with experimental SAXS data of the aptamer. A manual fit of the aptamer model onto a streptavidin crystal structure [12] showed that the aptamer could physically occupy two biotin-binding sites, and that it probably interacts with the loops that normally cover the biotin-binding pocket. This would explain the stoichiometry (2 aptamers per 1 streptavidin tetramer) and the presence of two distinct regions in the aptamer that are both essential for binding [12]. Although this modeling approach requires much more effort than standard secondary structure predictions, it can provide valuable information on the aptamer binding site and on the parts of the aptamer and the target protein that are involved in the interaction.

### 5.2. Experimental Approaches

As mentioned above, one of the requirements for the initiation of crystallization experiments with aptamer-protein complexes is establishing the minimal binding sequence of the aptamer. As described, this can be relatively easy if a recurring sequence or structural motif is found, but binding should still be confirmed.

Alternatively, a full length aptamer may be shortened from either the 5′ or 3′ end to various lengths, by either enzymatic trimming [63] or alkaline hydrolysis [13,64]. The resulting pool of fragments of randomly distributed lengths is incubated with the target, bound and unbound fragments are recovered as separate fractions, and run on a gel (e.g., polyacrylamide) to separate all individual fragments. From the patterns that emerge on the gel and the full-length sequence, the minimal binding sequence can be deduced. An alternative approach to determine the minimal sequence required for binding is the use of fragments of defined lengths obtained by PCR [15] or as synthetic constructs [14], and screening them for their binding capacity, e.g., by SPR. To get a better understanding of the aptamer-target interaction, nucleotides that are predicted to be important, for instance based on sequence or secondary and tertiary structure modeling, could be mutated to other nucleotides and their effect on target binding determined.

Secondary structure predictions are particularly helpful to identify the smallest DNA or RNA molecule still capable of binding the target, and may also reveal the nucleotides that are involved in target binding. On the other hand, it is also useful to obtain information on which regions or amino acids of the target are actually interacting with the aptamer. To this end, alanine scanning, replacing specific amino acids in a protein with alanine, has been successfully used to show that two arginine residues in the protease domain of non-structural protein 3 from the Hepatitis C virus are essential for aptamer binding [65]. Although successful, this method is very laborious. Hydrogen/deuterium exchange in combination with mass spectrometry (HXMS) [66] could provide an alternative method to map sites on the protein that interact with the aptamer. Other examples of the investigation of ligand specificity are the binding of biotin to an RNA aptamer. Several small molecules, similar to biotin, or fragments thereof, were tested for their ability to bind the RNA aptamer. The results suggest that binding is not enabled by a single functionality, but rather that several interactions of different parts of biotin are required simultaneously [13]. Results of another study, in which the binding capacity of various compounds to an ethanolamine-binding aptamer was investigated, showed that the aptamer has a preference for only one functionality in the target molecule: either an ethyl- or methylamine group [67].

Other advanced analytical techniques are available for investigating aptamer-target interactions. ITC is a method used to determine thermodynamic parameters of interactions; the affinity constant can, however, also be deduced from ITC data. SPR and QCM can be used to measure the association and dissociation rates that underlie the affinity constant of an aptamer-target interaction. SAXS can be applied to obtain information on the size and shape of macromolecular structures; like SAXS, DLS (also referred to as Quasi-elastic light scattering) can also be used for the determination of particle sizes and shapes, but it also provides information on the diffusion coefficients in solution. CD is particularly useful in determining secondary structures of proteins and aptamer-protein complexes. Combining these dedicated analytical techniques can provide useful insights into the aptamer target interaction. A recent example for which a combination of these techniques was used (ITC, DLS, NMR and SAXS) is the transformation of a cocaine binding aptamer to one that became specific for deoxycholic acid, by only replacing a single base pair [19].

## 6. Conclusions and Future Outlook

Co-crystal structures of aptamer-protein complexes have provided detailed information about the interaction between the nucleic acid aptamer and the protein, and can distinguish between electrostatic interactions [24] and hydrogen-bonding interactions [28]. Crystal structures are therefore crucial for a thorough understanding of the molecular basis of the interaction. Nevertheless, obtaining crystal structures of nucleic acid-protein complexes is challenging, because a large number of parameters can be varied and numerous conditions need to be screened. In the specific case of aptamer-protein complexes, screening becomes even more challenging, because care should be taken that the 3D-structure of the aptamer remains intact during crystallization experiments. Consequently, the nucleic acid parameters (e.g., length, presence or absence of sticky ends) are not easily varied because they could disrupt the 3D-structure of the aptamer, which should be avoided.

Despite the technical advances and the use of high-throughput crystallization methods, it will remain a major challenge to obtain aptamer-protein crystals. Improvements in NMR techniques will allow larger complexes to be analyzed at lower cost in the near future, providing an appealing alternative for crystallography. However, this requires improvements in data analysis routines, because data analysis becomes more complicated with increasing molecule size [68].

Alternative experimental approaches, such as alanine scanning, ITC, DLS, and SAXS are perhaps not as visually appealing as atomic resolution structures, but in combination with computational approaches, they may provide very useful information about the molecular basis of the interaction. In addition, some of these techniques can provide information on the dynamics of complex formation, whereas atomic resolution structures provide a time- and position-averaged image. One promising technique for obtaining high-resolution information on aptamer-protein interactions is cryo-electron microscopy (cryo-EM). Currently, cryo-EM is often used to study the structure of macromolecular assemblies, but its lower limit (~0.1 MDa) [69] is still too high for most of the aptamer-protein interactions described today. As technology advances, the lower limit might go down, and cryo-EM could become an attractive alternative for X-ray crystallography. Despite the many alternative methods to study aptamer-protein interactions, screening numerous crystallization conditions will remain, and for the time being, is the only way to obtain atomic resolution structures of aptamer-protein complexes.

## Supplementary Materials



## Figures and Tables

**Table 1:** Structures of aptamer-target complexes in the PDB database.

Target	DNA/RNA	PDB entry code	Ref.
**Aptamer-protein complexes**			

von Willebrand Factor Domain A1	DNA	3HXO 3HXQ	[20]
Alpha-thrombin (human)	DNA	1HUT	[21]
Alpha-thrombin (human)	DNA	1HAO 1HAP	[22]
Alpha-thrombin (human)	RNA	3DD2	[23]
Alpha-thrombin (human)	DNA	3QLP	[24]
NF-κB(p50)2	RNA	1OOA	[25]
NF-κB P50-RelB	DNA	2V2T	[26]
YmaH (Hfq)	RNA	3HSB 3AHU	[27]
a human IgG	RNA	3AGV	[28]
Enterobacterio phage MS2 coat protein complex	RNA	6MSF	[29]
Enterobacterio phage MS2	RNA	5MSF 7MSF	[30]
Enterobacterio phage MS2	RNA	1U1Y	[31]

**Aptamer-small molecule complexes**			

Malachite green	RNA	1F1T	[32]
Vitamin B12	RNA	1ET4 1DDY	[33]
Streptomycin	RNA	1NTB 1NTA	[34]
Biotin	RNA	1F27	[35]
