# Supplementary material for: Characterization of Aptamer-Protein Complexes by X-ray Crystallography and Alternative Approaches
Source: Int J Mol Sci. 2012 Aug 22;13(8):10537–52. doi: 10.3390/ijms130810537 (PMC3431876; doi:10.3390/ijms130810537)
Supplement: Supplementary file 1 [file ijms-13-10537-s001.pdf]

Supplemental Materials

Material and Methods

Crystallisation

Crystallisation experiments were performed using the sitting-drop vapour diffusion method with the conditions provided by the JCSG+ Suite (Qiagen), the Crystal screen, Crystal screen 2, and the Natrix and Natrix 2 crystallization screens (all from Hampton Research, CA, USA).

For the crystallisation experiments using the JCSG+ Suite, Crystal screen and Crystal screen 2 conditions, pure lyophilized streptavidin (M_r = 16.62 kDa; Leinco Technologies Inc., St. Louis, Mo, USA) was reconstituted in Milli-Q water to a concentration of 23.5 mg/mL (NanoDrop1000 spectrophotometer, Thermo Scientific, Wilmington, DE, USA), using a molar extinction coefficient at 280 nm of 41.940. The aptamer StrepApt5 (gggaACGCaccGATCGCAggtTtccc, Sigma; M_r = 7.96 kDa) was dissolved in Milli-Q water to a concentration of 1 mM. Streptavidin and StrepApt5 were mixed in a ratio of 1 tetramer: 5 aptamers, yielding a final protein concentration of 10 mg/mL. Drops consisting of equal volumes of reservoir solution (1 µL) and Streptavidin-StrepApt5 complex were equilibrated over 400 µL reservoirs (JCSG+) or 450 µL reservoirs (Crystal screen and Crystal screen 2) at 20 °C.

For crystallisation experiments using the Natrix and Natrix 2 crystallization screens, pure lyophilized streptavidin was reconstituted in Milli-Q water to a concentration of 25.5 mg/mL. StrepApt5 was dissolved in binding buffer (100 mM NaCl, 2 mM $MgCl_2$, 5 mM KCl, 1 mM $CaCl_2$ and 20 mM Tris/HCl, pH 7.6) to a concentration of 1 mM. Streptavidin and StrepApt5 were mixed in a ratio of 1 tetramer:2.2 aptamers, yielding a final protein concentration of 10 mg/mL. The mixture was left at room temperature to allow complex formation. In addition, samples with only streptavidin or StrepApt5 were prepared by diluting them in the same way as the Streptavidin-StrepApt5 sample. Drops consisting of equal volumes of reservoir solution (2 µL) and either Streptavidin-StrepApt5 complex, Streptavidin, or StrepApt5 were equilibrated over 450 µL reservoirs (JCSG+) at 20 °C.

X-ray Data Collection and Analysis

A crystal obtained using a reservoir solution consisting of 2.0 M ammonium sulphate and 5% *v*/*v* 2-propanol (Crystal screen 2 condition 5) was used for data collection (Figure 3). For cryoprotection, the crystal was soaked in reservoir solution containing 25% (*w*/*v*) glycerol. The crystal was mounted in a cryoloop and flash frozen in a stream of nitrogen gas at 110 K. Diffraction data was collected in house at 110 K using a Microstar rotating anode (Cu) X-ray source (Bruker AXS GmbH) in combination with Helios optics (Incoatec GmbH) and a MAR345dtb detector (Marresearch GmbH). In total, 220 data images were collected with an oscillation angle of 1°. The data was processed using the iMosflm program [1] and programs from the CCP4 Suite [2]. For data collection statistics see Table S1.

The structure of the crystallized protein was solved by molecular replacement using the program Phaser v.1.3 [3] with the native streptavidin structure (PDB code 3ry1; [4]) as the search model from which water and solvent molecules had been removed. After rigid-body refinement of the obtained solution, the R factor was 0.396. Further refinement was done with the program REFMAC5 [5], setting aside 5% of the reflections to monitor refinement progress with the R_{free} factor, but was stopped when it was clear that no aptamer was bound to the protein. Table S1 lists the refinement statistics.

Table S1. X-ray data collection and refinement statistics.

X-ray source	In house Microstar rotating anode X-ray source (Bruker AXS GmbH)		
Wavelength (Å)	1.5418 Å		
Space group	*C*2		
molecules/A.U	4		
Solvent content	36% (based on 64 kDa/tetramer)		
Unit-cell parameters			
a, b, c (Å)	83.9	47.6	104.7
α, β, γ (º)	90	100	90
Resolution (Å)	1.86	(1.96–1.86) *	
R_{merge}	0.101	(0.638)	
$R_{p.i.m}$	0.056	(0.338)	
Total No. of observations	138644	(18185)	
Total No. of unique reflections	32195	(4221)	
Mean $I/\sigma(I)$	7.7	(1.8)	
Completeness (%)	93.8	(85.3)	
Multiplicity	4.3	(4.3)	
**Refinement**			
R-factor	22.1		
R_{free}	27.1		
RMSD from target geometry			
Bond lengths (Å)	0.014		
Bond angles (°)	1.58		
Total number of atoms	3684		
Number of amino acids	475 (A: 16–136, B: 15–132, C: 15–133, D: 16–132)		
Number of water molecules	154		
Ramachandran analysis (%) [6]			
Core	88.8		
Allowed	10.3		
Generously Allowed	1.0		
Disallowed	0.0		

* Between brackets: Statistics for the highest resolution shell.

Table S2. Overview of crystals of streptavidin only and streptavidin-StrepApt5 complex in the same conditions.

Condition	Composition	Streptavidin only	Streptavidin-StrepApt5 complex
Natrix 1 25	0.08 M Magnesium acetate tetrahydrate, 0.05 M Sodium cacodylate trihydrate pH 6.5, 30% *w/v* Polyethylene glycol 4,000	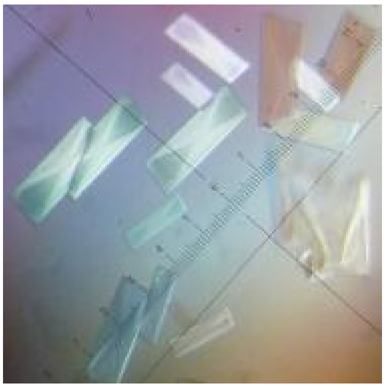	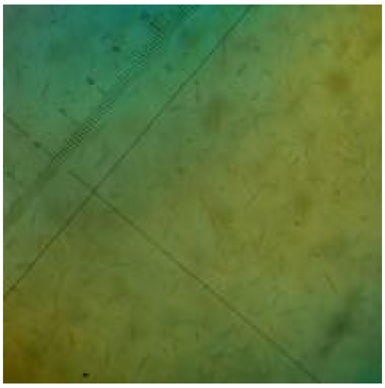
Natrix 1 27	0.2 M Ammonium acetate, 0.01 M Magnesium acetate tetrahydrate, 0.05 M Sodium cacodylate trihydrate pH 6.5, 30% *w/v* Polyethylene glycol 8,000	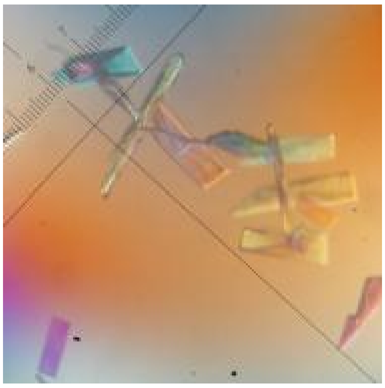	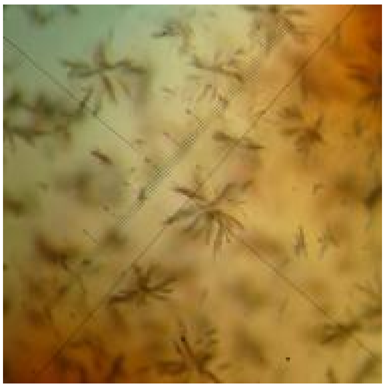
Natrix 1 45	0.025 M Magnesium sulfate hydrate, 0.05 M TRIS hydrochloride pH 8.5, 1.8 M Ammonium sulfate	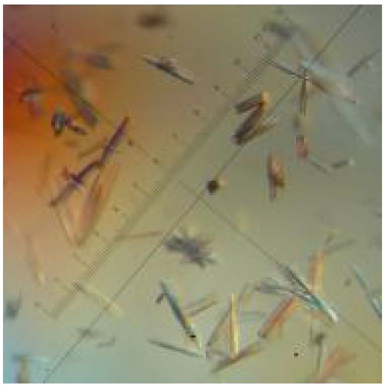	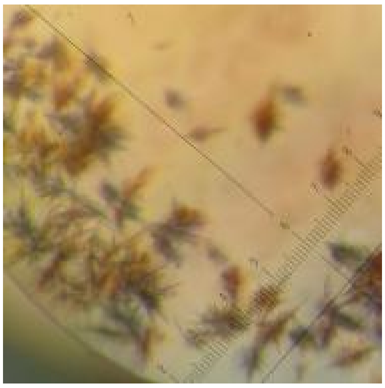
Natrix 1 48	0.2 M Ammonium chloride, 0.01 M Calcium chloride dihydrate, 0.05 M TRIS hydrochloride pH 8.5, 30% *w/v* Polyethylene glycol 4,000	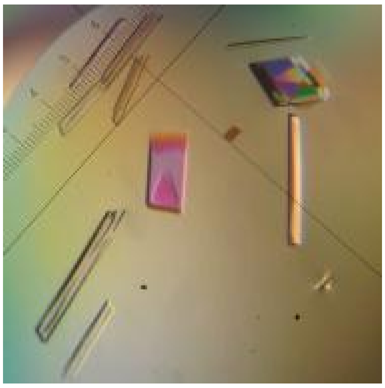	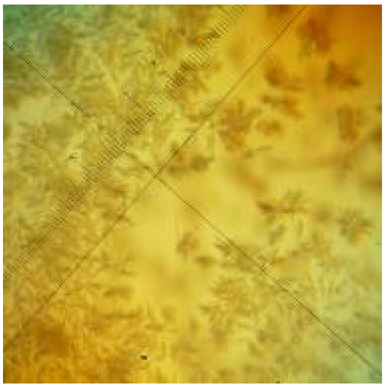

Figure S1. Sequence and secondary structure of StrepApt5.

GGGAACGCACCGATCGCAGGTTTCCC

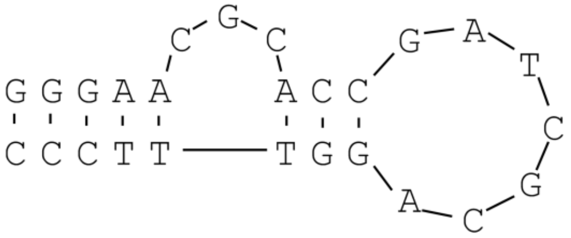

Figure S2. Structural analysis of streptavidin structure. (**A**) Electron density observed in the biotin-binding pocket of one of the subunits; density is present in all biotin-binding pockets; (**B**) The observed density is too small to have originated from an aptamer, but it neither corresponds to a full biotin molecule (shown in orange). We suspect it is a biotin analogue, used by the manufacturer to purify the protein. Unfortunately the resolution is not sufficient to identify the exact nature of this molecule; (**C**) Symmetry-related mates of the central streptavidin show that there is no physical space for a large DNA molecule. Biotins are shown as yellow spheres: 4 biotin molecules per streptavidin tetramer.

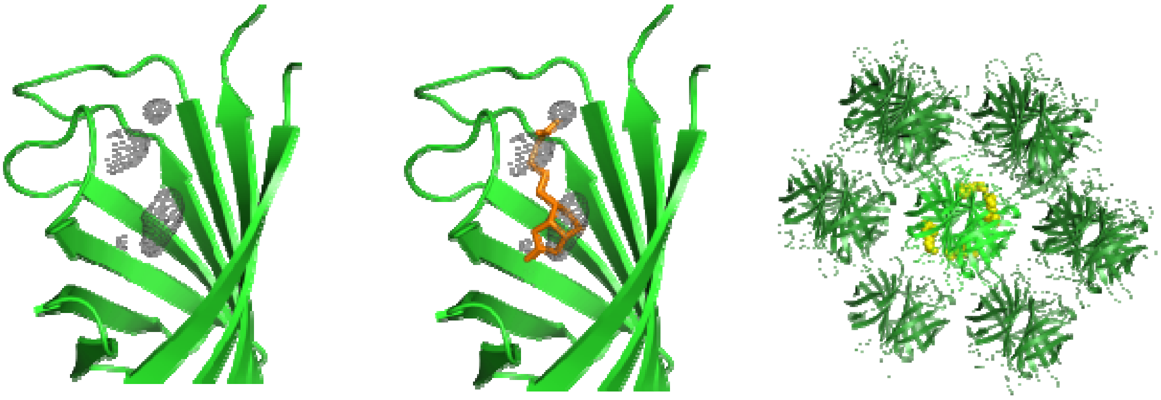

(**A**) (**B**) (**C**)

Figure S3. The structural model obtained from the collected data (unsuccessful attempt to obtain a streptavidin-aptamer structure) is shown in green. A biotin containing streptavidin structure (3MG5) [7] is shown in blue (biotins are hidden in this figure).

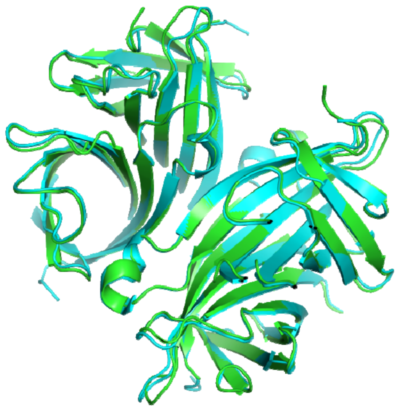

References

1. Battye, T.G.G.; Kontogiannis, L.; Johnson, O.; Powell, H.R.; Leslie, A.G.W. iMOSFLM: A new graphical interface for diffraction-image processing with MOSFLM. *Acta Crystallogr. Sect. D.* **2011**, *67*, 271–281.
2. Winn, M.D.; Ballard, C.C.; Cowtan, K.D.; Dodson, E.J.; Emsley, P.; Evans, P.R.; Keegan, R.M.; Krissinel, E.B.; Leslie, A.G.W.; McCoy, A.; *et al.* Overview of the CCP4 suite and current developments. *Acta Crystallogr. Sect. D* **2011**, *67*, 235–242.
3. McCoy, A.J.; Grosse-Kunstleve, R.W.; Adams, P.D.; Winn, M.D.; Storoni, L.C.; Read, R.J. Phaser crystallographic software. *J. Appl. Crystallogr.* **2007**, *40*, 658–674.
4. Le Trong, I.; Wang, Z.; Hyre, D.E.; Lybrand, T.P.; Stayton, P.S.; Stenkamp, R.E. Streptavidin and its biotin complex at atomic resolution. *Acta Crystallogr. Sect. D* **2011**, *67*, 813–821.
5. Murshudov, G.N.; Skubák, P.; Lebedev, A.A.; Pannu, N.S.; Steiner, R.A.; Nicholls, R.A.; Winn, M.D.; Long, F.; Vagin, A.A. REFMAC5 for the refinement of macromolecular crystal structures. *Acta Crystallogr. Sect. D* **2011**, *67*, 355–367.
6. Chen, V.B.; Arendall Iii, W.B.; Headd, J.J.; Keedy, D.A.; Immormino, R.M.; Kapral, G.J.; Murray, L.W.; Richardson, J.S.; Richardson, D.C. MolProbity: All-atom structure validation for macromolecular crystallography. *Acta Crystallogr. Sect. D* **2010**, *66*, 12–21.
7. Baugh, L.; Le Trong, I.; Cerutti, D.S.; Glich, S.; Stayton, P.S.; Stenkamp, R.E.; Lybrand, T.P. A distal point mutation in the streptavidin-biotin complex preserves structure but diminishes binding affinity: Experimental evidence of electronic polarization effects? *Biochemistry* **2010**, *49*, 4568–4570.

© 2012 by the authors; licensee MDPI, Basel, Switzerland. This article is an open access article distributed under the terms and conditions of the Creative Commons Attribution license (http://creativecommons.org/licenses/by/3.0/).
